# Supplementary material for: A Humanized Yeast Phenomic Model of Deoxycytidine Kinase to Predict Genetic Buffering of Nucleoside Analog Cytotoxicity
Source: Genes (Basel). 2019 Sep 30;10(10):770. doi: 10.3390/genes10100770 (PMC6826991; doi:10.3390/genes10100770)
Supplement: Supplementary file 1 [file genes-10-00770-s001.zip › Additional_Files/Additional_File7_GOTermHeatmaps.zip!/Additional_File7_GOTermHeatmaps/CORVET_complex.pdf]

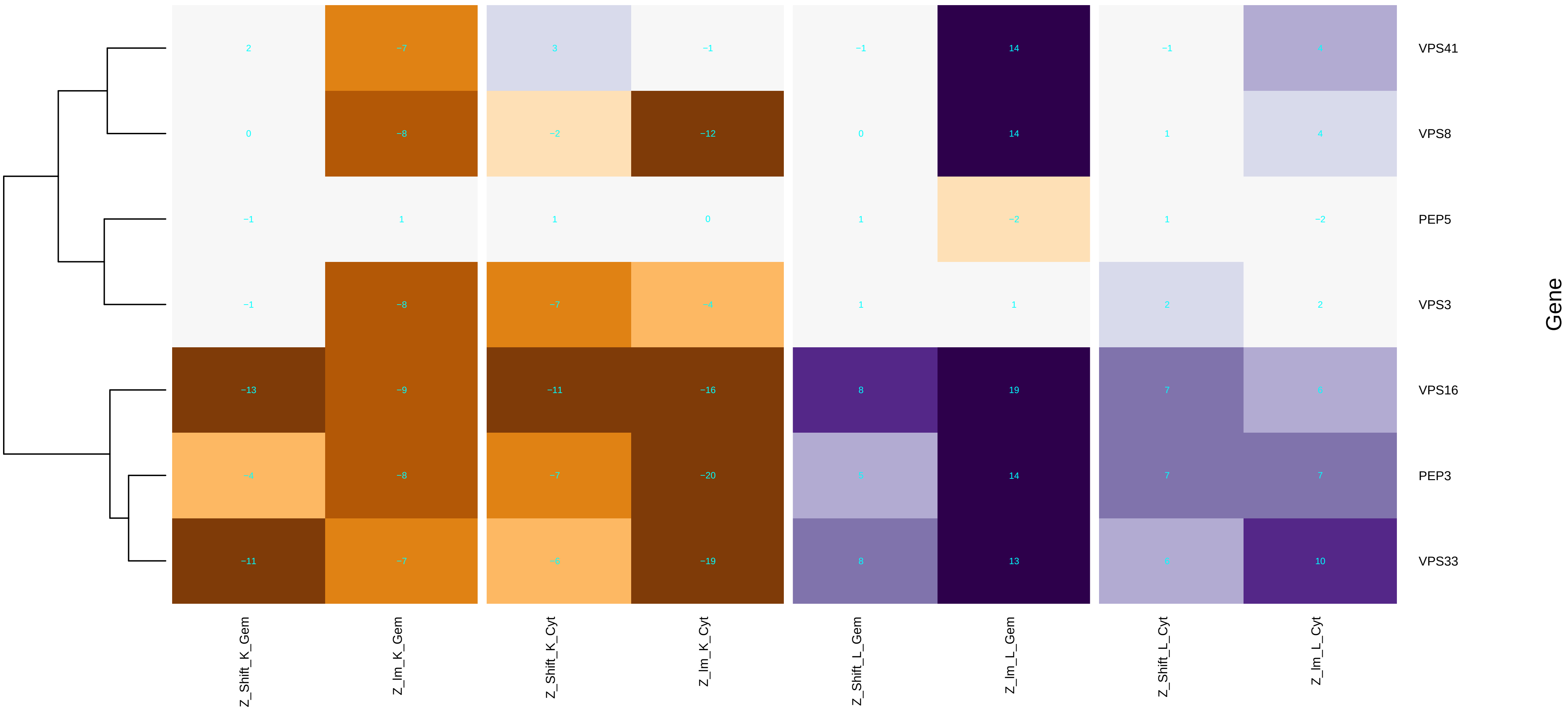

Gene
VPS41
VPS8
PEP5
VPS3
VPS16
PEP3
VPS33
2
−7
3
−1
−1
14
−1
4
0
−8
−2
−12
0
14
1
4
−1
1
1
0
1
−2
1
−2
−1
−8
−7
−4
1
1
2
2
−13
−9
−11
−16
8
19
7
6
−4
−8
−7
−20
5
14
7
7
−11
−7
−6
−19
8
13
6
10
Z_Shift_K_Gem
Z_Im_K_Gem
Z_Shift_K_Cyt
Z_Im_K_Cyt
Z_Shift_L_Gem
Z_Im_L_Gem
Z_Shift_L_Cyt
Z_Im_L_Cyt
